# Effect of provision of an integrated neonatal survival kit and early cognitive stimulation package by community health workers on developmental outcomes of infants in Kwale County, Kenya: study protocol for a cluster randomized trial

**DOI:** 10.1186/s12884-016-1042-5

**Published:** 2016-09-08

**Authors:** Lisa G. Pell, Diego G. Bassani, Lucy Nyaga, Isaac Njagi, Catherine Wanjiku, Thulasi Thiruchselvam, William Macharia, Ripudaman S. Minhas, Patricia Kitsao-Wekulo, Amyn Lakhani, Zulfiqar A. Bhutta, Robert Armstrong, Shaun K. Morris

**Affiliations:** 1 Centre for Global Child Health, The Hospital for Sick Children, Toronto, Canada; 2 Department of Community Health, Faculty of Health Sciences, Aga Khan University, Mombasa, Kenya; 3 Department of Paediatrics and Child Health, Aga Khan University Hospital, Nairobi, Kenya; 4 Department of Pediatrics, St Michael’s Hospital, University of Toronto, Toronto, Canada; 5 Institute for Human Development, Aga Khan University, Nairobi, Kenya; 6 Center of Excellence in Women and Child Health, Aga Khan University, Karachi, Pakistan; 7 Medical College, Faculty of Health Sciences, Aga Khan University, Nairobi, Kenya; 8 Department of Paediatrics, Faculty of Medicine, University of Toronto, Toronto, Canada; 9 Division of Infectious Diseases, The Hospital for Sick Children, Toronto, Canada

**Keywords:** Child development, Neonatal mortality, Kenya, Community health workers, Stimulation

## Abstract

**Background:**

Each year, more than 200 million children under the age of 5 years, almost all in low- and middle-income countries (LMICs), fail to achieve their developmental potential. Risk factors for compromised development often coexist and include inadequate cognitive stimulation, poverty, nutritional deficiencies, infection and complications of being born low birthweight and/or premature. Moreover, many of these risk factors are closely associated with newborn morbidity and mortality. As compromised development has significant implications on human capital, inexpensive and scalable interventions are urgently needed to promote neurodevelopment and reduce risk factors for impaired development.

**Method/Design:**

This cluster randomized trial aims at evaluating the impact of volunteer community health workers delivering either an integrated neonatal survival kit, an early stimulation package, or a combination of both interventions, to pregnant women during their third trimester of pregnancy, compared to the current standard of care in Kwale County, Kenya. The neonatal survival kit comprises a clean delivery kit (sterile blade, cord clamp, clean plastic sheet, surgical gloves and hand soap), sunflower oil emollient, chlorhexidine, ThermoSpot^TM^, Mylar infant sleeve, and a reusable instant heater. Community health workers are also equipped with a portable hand-held electric scale. The early cognitive stimulation package focuses on enhancing caregiver practices by teaching caregivers three key messages that comprise combining a gentle touch with making eye contact and talking to children, responsive feeding and caregiving, and singing. The primary outcome measure is child development at 12 months of age assessed with the Protocol for Child Monitoring (Infant and Toddler version). The main secondary outcome is newborn mortality.

**Discussion:**

This study will provide evidence on effectiveness of delivering an innovative neonatal survival kit and/or early stimulation package to pregnant women in Kwale County, Kenya. Study findings will help inform policy on the most appropriate interventions for promoting healthy brain development and reduction of newborn morbidity and mortality in Kenya and other similar settings.

**Trial registration:**

ClinicalTrial.gov NCT02208960 (August 1, 2014)

## Background

### Early childhood development

Over 200 million children under 5 years of age, the majority of whom reside in south Asia and sub-Saharan Africa, are failing to meet their development potential [1]. Children’s development encompasses several interconnected domains including cognitive, language, motor, social and emotional, and adaptive behaviour. Early child development (ECD) establishes the foundation for wellbeing and productivity throughout the life-cycle. For example, early cognitive impairment is a predictor of poor educational achievements later in life [1, 2]. Children who do poorly in school are less likely to become productive adults; they are more likely to have low incomes, high fertility, and have difficulty in providing adequate care for their children [1]. Thus, compromised development contributes to the propagation and exacerbation of poverty between generations and has implications on social equity and national development. In general, investments in women’s and children’s health yields high rates of return in health, social and economic benefits [3]. Notably, a recent economic simulation study that included 73 low- and middle-income countries (LMICs) estimated that by increasing preschool enrolment, only one component of ECD, by 25 % and 50 %, a total economic benefit of $10.6 and $33.7 billion, respectively, could be achieved with a benefit-to-cost ratio of 17.6 to 1 [4].

The first month of life is an important period in brain development during which there is significant neurogenesis, synaptogenesis, and myelination [1, 5]. Even small perturbations in the early developmental pathway of the brain can have long-term consequences on its strutural and functional capacity, and subsequent child development [5]. Brain development can be affected before and after delivery by both biological and psychosocial factors. Risk factors associated with compromised child development are complex and include poverty, malnutrition, infection, complications due to being born premature or with a low birthweight (LBW) and inadequate cognitive stimulation [6–8]. Since exposure to multiple risk factors increases the likelihood of poor development [9], the coexistence of factors associated with impaired development further exacerbates the problem in LMICs. The importance of promoting healthy brain development and preventing cognitive impairment in LMICs is further underscored by the absence of systems to identify and manage developmental delays soon after onset. Moreover, many risk factors for compromised development, namely infection and complications due to being born LBW and/or premature, are also closely related to newborn morbidity and mortality [10–13]. Newborn deaths account for approximately 40 % of all deaths in children under the age of five [14] and most are largely preventable. Thus, reducing the incidence of insults during the neonatal period has the potential to not only improve newborn survival but also improve long-term developmental outcomes.

A growing body of evidence suggests that early interventions can help prevent or reduce the loss of developmental potential in children [4, 15]. A recent systematic review reported substantial positive effects on child development in most effectiveness studies on ECD conducted in LMICs [4]. Interventions that target parenting and education support, preschool enrolment and programing, and improved maternal and child nutrition have each demonstrated positive effects on development [4, 15]. Interventions that were most effective were those that were comprehensive, engaged younger and disadvantaged children and families, were of adequate duration, intensity, and quality, and were integrated with other interventions including health, nutrition and conditional cash transfer programs [4, 15].

Parenting interventions include promotion of stimulation through responsive and developmentally suitable caregiver-child interactions. Stimulation has been shown to have positive effects on child development in numerous effectiveness studies and can be promoted through home visits, community groups, regular clinic visits, media outlets or through a combination of delivery mechanisms [4]. Delivery of stimulation interventions that focus on only the parent and family or on both the parent and the child has demonstrated positive effects. In rural Pakistan, children who received responsive stimulation delivered by Lady Health Workers, had significantly higher developmental scores on the cognitive, language, and motor scales at 12 and 24 months of age, and on the social-emotional scale at 12 months of age, than those who did not receive responsive stimulation [16]. Similarly, early stimulation among stunted children aged between 9 and 24 months in Kingston, Jamaica had a beneficial and sustained effect on their development at 2 years of age and during adolescence [17, 18]. Despite evidence on the effectiveness of stimulation interventions in early childhood, successful scale-up of programs to sustainably improve child development has been variable [4]. In addition, there are limited data on the effect of parenting interventions that focus on the engagement of pregnant women. Inclusion of pregnant women in early stimulation interventions has shown promise [19] and has been identified as a research area that merits further exploration [4].

Although a relationship between newborn morbidities and impaired cognitive development [6, 10, 20, 21] has been established, there remains a paucity of data on how interventions, that either reduce newborn maladies or facilitate their early detection, impact child development. For example, while numerous controlled studies have reported an association between emollient therapy and reduced risk of infection, improved thermoregulation and growth [22], and reduced newborn mortality in preterm neonates [23], neurodevelopmental outcomes have only been reported in one emollient therapy trial [24]. Of note, the study was limited to assessment of neurobehavioral aspects during the neonatal period and demonstrated no difference between intervention and control groups [24]. In addition, while umbilical cord cleansing with chlorhexidine has been shown to reduce both neonatal mortality and omphalitis/infections [25]; to the best of our knowledge, no studies have explored the effect of chlorhexidine application on developmental outcomes. While several studies have investigated the beneficial effects of Kangaroo Mother Care (KMC) on morbidity and mortality among LBW infants, limited data are available on the association between KMC and long-term developmental outcomes [26, 27]. Given the link between neonatal insults and compromised development, the importance of interventions for the prevention and early detection of newborn illness goes well beyond their effect on short-term health outcomes and newborn survival.

### Child health and development in Kenya

As is the case in most LMICs, Kenya has no data on child development. Using poverty and stunting (height-for-age z-scores below −2 SD from the median of the reference population) as indicators of poor development, the prevalence of disadvantaged children under the age of five in Kenya in 2004 was estimated to be between 40 and 60 % [1].

Stunting in early childhood is caused by poor nutrition, which in itself is often aggravated by infection. Significant associations have also been reported between stunting and developmental delays [7]. Kenya was ranked 47^th^ out of 136 countries with highest prevalence of stunting in 2009 [28]. According to the 2014 Kenyan Demographic Health Survey (KDHS), approximately 25 % of Kenyan children are stunted, while 8 % are severely stunted (height-for age z-scores are below −3 SD from the median of the reference population) [29]. Stunting was noted to be more common among children in rural areas (29 %) compared to those in urban areas (20 %). An inverse relationship was also found between household wealth and child stunting. Stunting in Kenyan children decreases with rising household wealth [29]. In Kenya, associations between height and child development measures [30] as well as socioeconomic status during infancy and cognition at 5 years of age have also been reported [31].

Child mortality rates provide a basic indicator of a country’s socioeconomic level and quality of life. During the 5-year period prior to the 2014 KDHS survey, child, infant and newborn mortality rates were 52, 39 and 22 per 1000 live births, respectively [29], falling short of the country’s Millennium Development Goal 4 (MDG4) targets [32]. While Kenya has experienced a downward trend in under-5 mortality rates since 1998, newborn mortality rates have declined much more slowly. Today, newborn mortality accounts for approximately 40 % of all deaths within the first 5 years of life in Kenya [29].

### Kenyan health system

The Constitution of Kenya, 2010, created a decentralized system of governance that became operational in 2013. In the decentralized structure, selected functions including provision of health care services were devolved to 47 County level governments; County governments are responsible for health facilities, pharmacies, ambulance services, and the promotion of primary health care in their counties [33]. Health service delivery is structured along six tiers of service provision that range from level-1 (community) to level-6 (tertiary referral facilities) [33]. Community-based health services, which comprise basic promotive, preventive and simple curative health, aim to strengthen primary health care at the community level. The building block for level-1 health service provision is the Community Unit where each unit comprises between 5000 and 10,000 people, and served by 50 to 75 community health workers (CHWs) and 1 or 2 community health extension workers (CHEWs). CHEWs are formally engaged, government-paid employees of the health system who, among other tasks, are responsible for providing continuous training and supervision to approximately 50 CHWs. This implies that each CHEW supervises approximately 25 CHWs. CHWs are part-time volunteer workers who act as ‘gate keepers’ of health in the community and are each responsible for providing care to about 20 households. CHWs are connected to primary health facilities through their supervising CHEW. While the existing Community Health Strategy (CHS) is appreciated, high attrition rates, lack of accountability for volunteer CHWs and lack of funds to pay CHW salaries need to be addressed [34].

### Rationale

In order for children to reach their developmental potential, interventions designed to promote development, alongside the prevention and early detection of newborn illness are essential [35]. This study aims to investigate the impact of delivering a neonatal survival kit and/or early cognitive stimulation package, on development at 12 months of age and neonatal mortality.

## Methods/Design

### Objective and hypothesis

The primary aim of this study is to determine whether an integrated neonatal survival kit and/or newborn stimulation package, when delivered to pregnant women during their third trimester of pregnancy by volunteer CHWs, improves developmental outcomes at 12 months of age. Through a reduction in severe infections and improved thermoregulatory care in the first month of life, we hypothesize that utilization of the neonatal survival kit will result in an improvement of at least one standard deviation in development at 12 months of age as measured by the Protocol for Child Monitoring – Infant/Toddler version (PCM-IT) assessment. Moreover, we hypothesize that the combination of both the neonatal survival kit and early stimulation messaging will have an additive effect on development. Secondary objectives are: i) to determine if introduction of the neonatal survival kit reduces all-cause neonatal mortality; ii) explore whether use of the kit is effective in reducing the incidence of omphalitis and/or severe infection, improves caregiver ability to identify hypothermia and/or fever through use of ThermoSpot, and improves identification of low birth weight through use of a handheld scale. The study will also determine if the interventions increase appropriate referral to local health facilities. Finally, the study will assess knowledge, attitudes, and practices of mothers with their newborn and their willingness to use the kit and their capacity to pay for it.

### Study setting

**Fig. 1:**
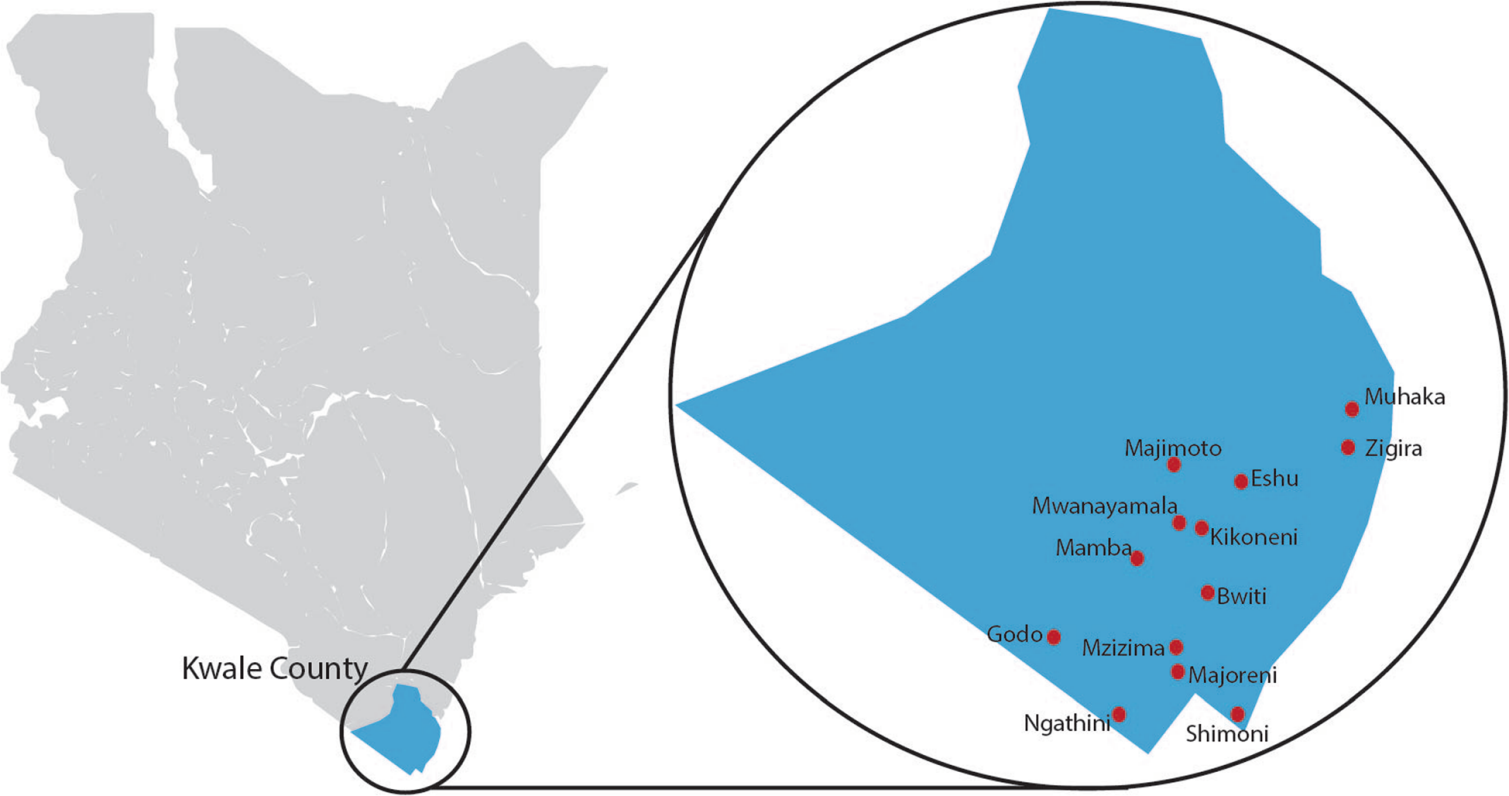
Map of the study catchment area in Kwale County, Kenya. The map image was adapted with permission from https://commons.wikimedia.org/w/index.php?curid=28868036. By Nairobi123 - Own work, CC BY-SA 3.0

This study is being conducted in Kwale County, which is located in the southern most point of Kenya in the former Coast Province (Fig. 1). Kwale County covers an area of 8270.2 square km, has a total population of 649,931 [36] and is divided into 4 sub-counties namely, Kinango, Lunga Lunga, Matuga and Msambweni. The average temperature in Kwale County is 24.2 °C and the region experiences both dry and wet seasons; the estimated precipitation levels are between 400 mm and 1680 mm per year.

Within Kwale County, there are 3 government hospitals, 2 private hospitals, 8 health centres and 64 dispensaries; the average distance covered to reach the nearest health facility is 7 km [36]. Vast distances, a poor network of roads and limited access to motorized transportation for many make it difficult to access health care facilities. In spite of government emphasis on the promotion of facility deliveries, more than 60 % of women in Kwale County still deliver at home without the assistance of skilled birth attendants [37]. The estimated under-five mortality rate in Kwale County, 149 deaths per 1000 live births, is higher than the national average, 116 deaths per 1000 live births [36]. Similarly, neonatal mortality rates in the rural areas of Kwale County are believed to be approximately double the national average of 22 per 1000 live births [29].

In this trial, participant recruitment, informed consent, newborn enrolment and data collection take place in the rural communities surrounding 13 dispensaries in Kwale County (Fig. 1). The average catchment population surrounding each dispensary is ~7500 individuals.

### Design and cluster definition

**Fig. 2:**
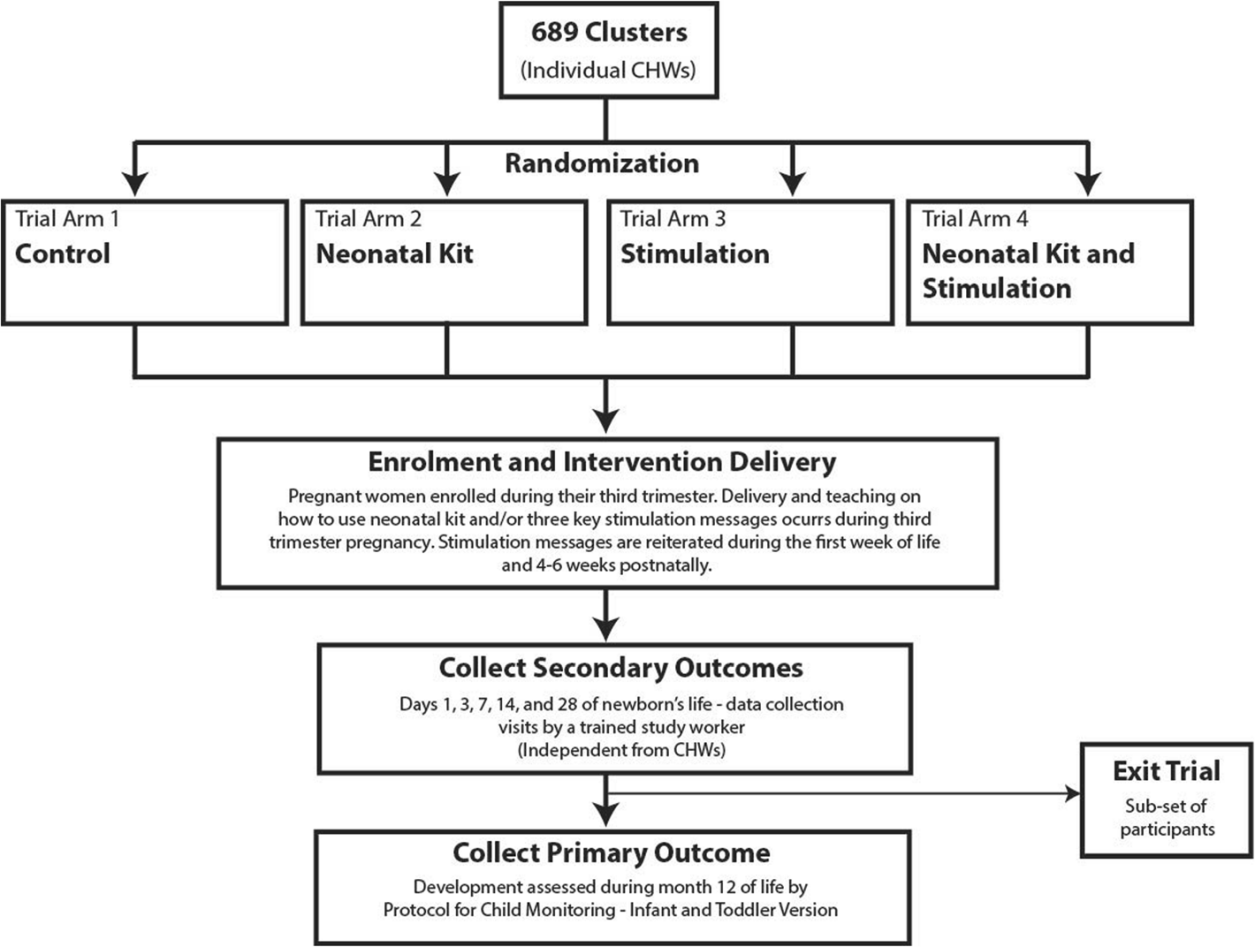
Schematic diagram of trial activities

This community-based study is a cluster randomized controlled, pragmatic, open label intervention trial with four trial arms: neonatal survival kit; stimulation messaging; combination of neonatal survival kit with stimulation messaging; and control (current standard of care in Kwale County [38]) (Fig. 2). A cluster is defined as a single CHW, where each CHW is responsible for the provision of basic health promotion and disease prevention services to approximately 20 households. Within one village, the distance between homes covered by one CHW can be large. Geographic distance thus minimizes any unintended diffusion of arms of interventions between households, especially those serviced by different CHWs (clusters).

### Interventions and comparator

To facilitate future scale-up efforts, the delivery of each intervention was built into the country’s existing health system infrastructure. CHWs routinely conduct home visits to pregnant women during their third trimester of pregnancy, and post-delivery during the first week of life and again 4–6 weeks post delivery. Specifically, the timing of intervention delivery coincides with the existing schedule of CHW home visits. In this trial, a trained study worker accompanies CHWs to their third trimester home visit, explains the study to potential participants, and seeks informed written consent. After obtaining consent, CHWs deliver the appropriate intervention(s) and corresponding education to enrolled pregnant women. In control clusters, CHWs deliver the current standard of community-based pre-natal care in Kenya [38].

**Fig. 3:**
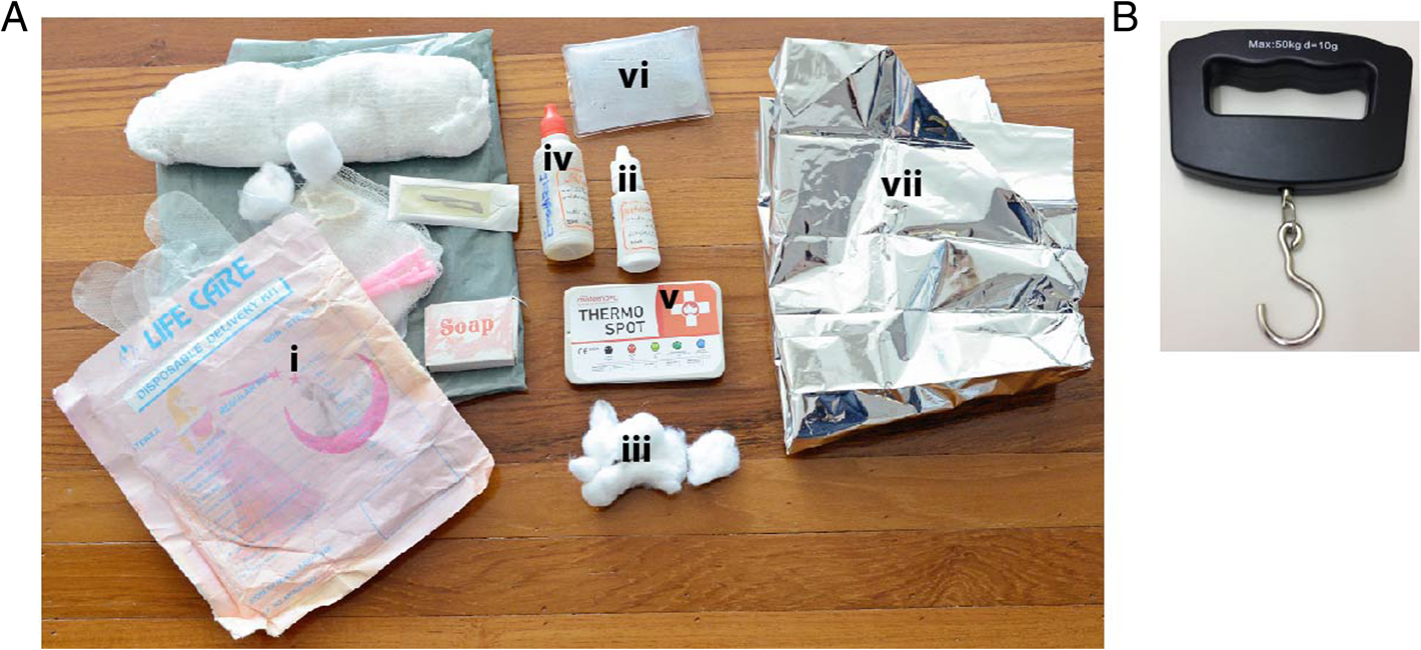
Integrated neonatal survival kit contents. **a** The neonatal survival kit includes: i) clean delivery kit, ii) 4 % chlorhexidine solution that is to be applied with iii) cotton balls, iv) sunflower oil emollient, v) ThermoSpot, vi) a reusable instant heat pack, and vii) a Mylar infant blanket. **b** A handheld battery operated weighing scale will be provided to CHWs in the intervention cluster

The integrated neonatal survival kit comprises a clean birth kit, 4 % chlorhexidine solution, sunflower oil emollient, ThermoSpot™, Mylar infant sleeve, and a reusable, instant heat pack (Fig. 3). In addition, CHWs in the intervention groups are also supplied with a handheld battery-operated weighing scale with a suspended sling to weigh newborns (Fig. 3). A detailed description of each kit component, its intended utilization and associated evidence has been published elsewhere [39].

The newborn cognitive stimulation program focuses on teaching three key messages to enhance the caregivers’ current practices including: making eye contact and talking to their children; engaging in responsive feeding and caregiving; and singing songs, including those with gentle touch (Table 1). The cognitive stimulation program was adapted from the UNICEF and WHO Care for Child Development package [40], which promotes the appropriate use of communication and play activities by guiding caregivers to be sensitive and responsive to signals from their child. CHWs teach caregivers to integrate each stimulation message into daily activities (e.g., during feeding, bathing, bedtime routines). Three fifteen-minute sessions take place in the homes of participants. At the first visit during the third trimester visit, caregivers are given a locally developed one-page pictorial brochure to act as a memory aid. CHWs describe the messages verbally as participants follow along with the pictorial illustrations of behaviors associated with each of the key messages. The information is re-iterated by CHWs at subsequent routine visits during the first week of life, and at the four to six week follow-up visit. At post-natal visits, CHWs provide modeling, coaching and support while encouraging caregivers to practice the behaviors associated with the three key messages.

**Table 1:** Cognitive stimulation messages

Key message
Making eye contact and talking to their children • Encourage caregivers to look into baby’s eyes, smile and talk/sing to their baby • Provide ways for child to see, hear, move their arms and legs freely, and touch (e.g., Caregivers are taught to slowly move colorful objects in front of the child) • Caregivers are taught to not cover the child’s face for long periods of time
Responsive feeding and caregiving
• Teach caregivers that children learn to communicate their needs through movements, sounds and cries. For example, children show interest in breastfeeding by becoming fussy, sucking their hand, or moving their heads toward the breast. • Caregivers are taught to emulate the child’s movements and sounds. Copying the child’s sounds and movements helps caregivers pay closer attention to the child. By imitating the child’s sounds, or movements, the child will often then repeat the activity, in order to get the caregiver to respond again. This increases the child’s attempts to make sounds and move, and the caregiver’s pleasure.
Singing songs, including those with gentle touch
• Encourage the use of core songs based on cultural practices and accompany each by gentle touch • Caregivers are taught that skin-to-skin contact is good and they are encouraged to gently soothe, stroke and hold the child

### Sample size and power estimation

Based on an estimated live birth rate of 39 per 1000 population, baseline NMR of 44 per 1000 live births, and approximately 5 live births per cluster (personal communication Amyn Lakhani), the randomization of 689 CHWs (clusters), with a 25 % chance of being randomized to any one of the four groups (neonatal survival kit, stimulation, neonatal survival kit and stimulation arm, and control), with 90 % enrollment of eligible mothers and including up to a 10 % loss to follow up will provide >90 % power to detect a one standard deviation difference in PCM-IT scores and >80 % power to detect a 40 % reduction in mortality between any two of the four groups. To ensure that randomization was unbiased, a scientist who is not directly involved in the research project performed the cluster randomization. CHWs were trained on the delivery of their randomly assigned intervention at a 1-day intervention-specific workshop; training included specific instructions for CHWs to restrain from sharing their intervention- or trial-specific knowledge with individuals outside their assigned households.

All pregnant women who intend to give birth and permanently reside in the trial catchment area for at least 12 months after delivery and their home or facility-born live births are eligible for enrollment in this study. Enrolment will take place over a period of 12 months targeting a final sample size of 3000 newborn-mother pairs. The first pregnant woman was recruited on November 24^th^, 2014 and the first newborn enrolled on November 28^th^, 2014.

### Study outcome measures

Developmental outcomes will be assessed using the PCM-IT in a subset of participants at 12 months of age. The PCM-IT combines both parental report and direct observation to provide a comprehensive evaluation of children’s motor skills, cognition, language, personal and socio-emotional development (Table 2). The PCM-IT assessment was derived from infant assessment tools that were developed and standardized in Kenya including the Kilifi Developmental Inventory (KDI) [41], the Developmental Milestones Checklist, Version 2 (DMC-II) [42, 43] and the Profile of Social-Emotional Development (PSED). The PSED is based in part on the Brief Infant/Toddler Social Emotional Assessment [44] to assess social cognition, independence, emotional lability, maladaptive behavior, and social competence [45]. Items for the DMC were drawn from several published measures, including the Griffiths Mental Developmental Scale for Infants [46] and the Vineland Adaptive Behavior Scale [47].

**Table 2:** Protocol for child monitoring assessment

Developmental domain	
Motor	• Evaluation of gross and fine motor skills • Assessed through direct observation and parental report • Example of items/activities that are directly observed: o Head control o Lifting upper body o Reaches out for moving object • Example of items/activities that are evaluated through maternal report: o Sits with/without support o Stands with/without support o Crawls/walks
Cognition	• Evaluation of cognition and executive function subscale • Executive function subscale made up of two tasks: self-control and A-not-B tasks • Assessed through direct and discreet observation • Example of items/activities: o Stacking cubes o Removing cubes from a container o Matching colours o Discretely monitor time until child reaches for desirable object they’ve been asked not to touch
Language	• Evaluation of language development through maternal report or if possible, direct observation • Example of items evaluated: o Repeating strings of vowels o Understands/uses gestures o Understands/uses words
Self help/Adaptive Behaviour	• Independence and adaptive behaviour assessed through maternal report • Example of items evaluated: o Dresses self o Feeding behaviour o Indicates when wet
Social Emotional	• Social items and emotional regulation assessed through maternal report • Example of Social items evaluated: o Recognition of people o Reaction to own name • Example of emotional regulation items evaluated: o Eating habits o Playing behaviour

For the majority of items on the PCM-IT, the child is evaluated on a 4-point rating scheme from ‘0’ to ‘3’ (‘0’ denotes ‘not able’; ‘1’ denotes ‘able to carry out the activity momentarily’; ‘2’ denotes ‘carries out the activity but with limitations’; and, ‘3’ denotes ‘able to carry out the activity with little effort’). For all items related to emotional regulation, frequency of occurrence is scored on a 3-point Likert scale (‘0’denotes ‘never or rarely happens’; ‘1’ denotes ‘happens sometimes’; ‘2’ denotes ‘always happens or most of the time’). The first 12-month PCM-IT assessment is scheduled to take place in November 2015.

Secondary outcomes include all-cause newborn mortality (death within the first 28 days of life), the incidence of omphalitis, severe infection, identified cases of hypothermia and hyperthermia, number of LBW newborns identified, health facility usage, and knowledge, attitude, practice and willingness to pay for neonatal kit (Table 3). Due to the nature of the intervention, blinding was not possible. However, to reduce measurement bias, an independent team that is not involved in the delivery of the intervention collects outcome data.

**Table 3:** Primary and secondary outcome measures

Outcome	Definition
Primary Outcome	
Neurodevelopment at 12 months of age	Defined by the development score assigned at 12 months of age as measured by the standardized Protocol for Child Monitoring – Infant and Toddler Version
Secondary Outcomes	
All-cause neonatal mortality	Death from any cause in the first 28 days of life
Incidence of omphalitis	A) None (no redness or swelling); B) Mild (inflammation limited to the cord stump); C) Moderate (inflammation extending to the skin at the base of the cord stump less than 2 cm); or D) Severe (inflammation extending more than 2 cm from the cord stump)
Incidence of severe infection	A) Convulsions; or B) Fast breathing (60 breaths per minute or more); or C) Severe chest indrawing; or D) Movement only when stimulated or no movement at all; or E) Not feeding at all for at least 12 h
Cases of hypothermia and hyperthermia identified	Defined using ThermoSpot: A) Moderate hypothermia: pale green and red face (35 °C to 36 °C) B) Severe hypothermia: black face (<35 °C) C) Hyperthermia: blue face (>=39 °C)
Number of LBW newborns identified	<2500 g at first weighing
Health facility use	Health centre visits to A) Dispensary B) Health Centre C) Subcounty Hospital D) County Hospital E) Coast General Hospital F) Private clinic/hospital
Knowledge, attitudes, practices and willingness to pay for the newborn toolkit	Defined by self-reported data on: A) Use of each kit component B) Purpose of each kit component C) Perceptions on ease of use D) Perceptions on safety and effectiveness of kit E) Willingness to Pay for the kit

### Data collection and data management

Forty data collectors, each with a minimum qualification of high school education level, underwent 3 days of formal training. In this training, the goals of the study were explained to them, they were taught basic methodology, and provided with an in-depth understanding of each data collection form. To assess secondary outcomes, data collectors visit homes in the intervention and control clusters on days 1 (or as soon as possible after birth notification), 3, 7, 14, and 28 of life. Data collection in the first few days of a newborn’s life is critical to the study’s success as many of the secondary outcomes measured in this study may occur very soon after delivery. Data collection visits are considered missed once the next data collection point is reached. For example, the day 7 visit can be completed up to day 13; on day 14, the day 7 visit is considered missed. The day 28 visit is considered missed, if two weeks after the scheduled visit, the questionnaire has still not been completed. To facilitate timely birth notifications, at the time of consent, participants are provided with contact information and instructions to notify the study team and/or their CHWs as soon possible following delivery. Moreover, a small monetary incentive is provided to both participants and CHWs for birth notifications that lead to the enrolment of an infant by day three of life.

At the first data collection visit, the data collector administers a questionnaire to document the events surrounding delivery and the immediate post-natal status of the newborn, including measurement of newborn weight. At each subsequent visit, data collectors administer a questionnaire covering the events that have occurred since the last visit to assess outcomes in the newborn. Data collectors are not trained to treat outcome conditions; rather, they are trained to identify newborn danger signs and make appropriate referrals. As an extra precaution, data collectors are required to report all newborn danger sign to their direct supervisor who then relays the information to the CHEWs based at their dispensary. On the day 28 visit, data collectors also administer a brief questionnaire to participants enrolled in the kit-only and kit plus stimulation intervention arms to assess participants’ knowledge, attitudes and practice toward the neonatal kit as well as their willingness to pay for the kit. Information on caregiver socio-demographics characteristics is also collected.

If a newborn is not enrolled into the study by day three of life, they are no longer eligible to receive a complete series of data collection visits (days 1, 3, 7, 14, and 28 of life). In these situations, a short questionnaire is administered on day 28 of life to capture information on the pregnancy outcome (i.e., live birth or stillbirth), whether components of the kit were used by participants in the kit-only and kit plus stimulation groups, and in cases of live births, the neonatal outcome (i.e., alive or dead on day 28 of life). In cases where the study team is not aware of the birth of a baby until after day 28 of life, a data collector is dispatched to the home of the participant as soon as the delivery notification is received. If the mother or caregiver is unavailable at the time of this visit, the data collection visit is rescheduled to the earliest available time.

In the event of a newborn death, the parent will be given the choice of participating in a verbal autopsy [13]; a minimum 2-week mourning period precedes the administration of the verbal autopsy. To determine cause of death, two pediatricians will review all completed verbal autopsy forms. If a discrepancy exists in the cause of death ascertained by the two primary reviewers, a third senior pediatrician will act as an adjudicator.

At 12 months of age, a subset of children will undergo a standardized assessment of development by a study worker trained in the delivery of the PCM-IT assessment. The study worker will be blinded to the arm of the trial to which the participant was randomized. The assessment will take place in the child’s home in the presence of their primary caregivers and will take approximately 1 h to administer. At the assessment, the study worker will explain and demonstrate each new task before monitoring the child’s attempt at the activity and scoring their abilities. A number of items on the PCM-IT, for example ‘sitting with support,’ will be scored through observing the child’s behavior. Children will receive a score for all 57 assessment items in the checklist and a summated score will be calculated for four areas; gross and fine motor skills, cognition and executive function. In addition, the parental interview covering 46 items will yield scores in four areas: language, self-help/adaptive, social and emotional regulation. The PCM-IT assessment will also be used to measure the baseline developmental score within the study population in a group of children born before this study commenced and thus were not exposed to the kit, stimulation, or other study activities. A questionnaire will be administered to every study participant who receives a developmental assessment to assess various demographic and socioeconomic factors, which may be associated with developmental outcomes.

Data are collected on paper forms in the homes of participants. Completed data forms are transported to local health facilities where they are stored securely until they can be transported to the centralized Data Management Unit (DMU) in Mombasa, Kenya. Each week, paper forms are delivered to the DMU where they are double entered into electronic format by two independent data clerks. Prior to data entry, all forms are checked for completeness and consistency. In case of inconsistency or missing responses, the editors flag the errors/omissions and consult the interviewers for possible explanations. For data entry, databases and entry screens were developed using EpiInfo version 3.5.1. The entry screens employ range and consistency checks to minimize entry of erroneous data.

### Data analysis plan

Data will be analyzed as intention to treat using univariate and multivariate methods in STATA version 13. PCM-IT scores will be compared using Student’s *t*-test and linear regression models will be used to control for confounders. Survival and other related outcomes in the four groups will be analyzed using Cox proportional hazards analysis. All estimates will be adjusted for cluster allocation according to generalized estimating equation method.

## Discussion

Awareness of the importance of child development is increasing in LMICs. Interventions that aim to promote early child development alongside the prevention and timely detection of newborn illness are important investments in minimizing the loss of human capital [35]. While evidence exists in support of various interventions and delivery mechanisms to promote child development, at present, very few studies have explored the feasibility and effectiveness of volunteer community health workers delivering interventions that aim to promote development and/or reduce newborn morbidity and mortality directly to women during pregnancy. The utilization of CHWs to deliver and provide education regarding the interventions has the potential to positively influence the health of the community and will facilitate national scale-up and long-term sustainability. Indeed, the effectiveness of CHWs in delivering key health promotion messages has been previously demonstrated [48]. The findings of this trial will provide evidence to inform policy on the implementation of sustainable interventions in rural settings to promote child development and reduce risk factors of compromised development, newborn morbidity and mortality.

### Trial status

Enrolment in ongoing

## Abbreviations

CHEW: Community health extension worker
CHS: Community health strategy
CHW: Community health worker
DMC: Developmental milestones checklist
DMU: Data management unit
ECD: Early childhood development
KDHS: Kenya demographic and health survey
KDI: Kilifi developmental inventory
KMC: Kangaroo mother care
LBW: Low birth weight
LMIC: Low and middle income country
MDG4: Millennium development goal 4
PCM-IT: Protocol for child monitoring – infant and toddler version
PSED: Profile for socio-emotional development
